# Pembrolizumab Therapy Triggering an Exacerbation of Preexisting Autoimmune Disease: A Report of 2 Patient Cases

**DOI:** 10.1177/2324709616674316

**Published:** 2016-10-25

**Authors:** Sneha D. Phadke, Ramez Ghabour, Brian L. Swick, Andrea Swenson, Mohammed Milhem, Yousef Zakharia

**Affiliations:** 1 Department of Internal Medicine, Divison of Hematology/Oncology, University of Iowa Hospitals & Clinics, Iowa City, IA, USA; 2 Department of Internal Medicine, University of Iowa Hospitals & Clinics, Iowa City, IA, USA; 3 Department of Dermatology, University of Iowa Hospitals & Clinics, Iowa City, IA, USA; 4 Department of Neurology, University of Iowa Hospitals & Clinics, Iowa City, IA, USA

**Keywords:** autoimmune, melanoma, immunotherapy, psoriasis, myasthenia gravis

## Abstract

Historically, metastatic melanoma was uniformly and rapidly lethal, and treatment options were limited. In recent years, however, checkpoint inhibitors have emerged as an accepted standard treatment for patients with advanced melanoma. In clinical trials, these agents have been largely well tolerated and have the potential to result in durable responses. Importantly though, one must recognize the unique side effect profile of these therapies, which can trigger or exacerbate underlying autoimmune disease. Whether this autoimmune activation is associated with a clinical response to therapy has been debated, and while not definitive, there is evidence in the literature of a possible association. The 2 cases presented describe this autoimmune phenomenon, along with a review of the existing literature on the relationship between response to immunotherapy and autoimmune side effects.

## Introduction

Immunotherapy using interleukin-2 (IL-2) has long been a part of our historically small armamentarium against advanced melanoma. Treatment with IL-2 was largely limited to patients who could tolerate the potentially serious adverse effects, and while only a minority of patients obtained benefit, those who did experienced prolonged response durations.^1-3^ Interferon has also been studied in the metastatic setting, and while it showed modest efficacy, durations of response were short and toxicities potentially prohibitive.^4,5^ Therapeutic options are now increasing with the emergence of checkpoint inhibitors, which are shifting the way that oncologists view a once universally lethal disease. Although checkpoint inhibitors are relatively well tolerated, some patients will experience immunologic side effects, known as immune-related adverse events (irAE). Additionally, those with preexisting autoimmune disorders may experience flares of their disease. Here we present 2 patients with metastatic melanoma treated with the PD-1 checkpoint inhibitor pembrolizumab who developed exacerbations of preexisting autoimmune disease.

## Case 1

I.M. is a 67-year-old Caucasian man with a history of noninvasive low-grade (TaLG) bladder cancer diagnosed in 2013, treated with transurethral resection. Two years later, on a surveillance cystoscopy, he was found to have lesions at the posterior bladder dome and within the prostatic fossa. Biopsies of the lesions revealed malignant melanoma, BRAF V600E positive. The patient denied any history of melanoma and had no skin lesions suspicious for a primary melanoma. A review of the bladder pathology from 2013 confirmed papillary bladder cancer with no evidence of melanoma. Staging positron emission tomography/computed tomography (PET/CT) scan revealed scattered osseous lesions as well as right hilar and mediastinal lymphadenopathy. A brain magnetic resonance imaging (MRI) was negative for intracranial metastases. Other pertinent medical history included psoriasis and psoriatic arthritis, which had been well controlled on a weekly dose of 12.5 mg of oral methotrexate. After careful discussion with the patient about the potential side effects of treatment including worsening of psoriasis, methotrexate was discontinued and pembrolizumab was initiated at 2 mg/kg every 3 weeks. Three doses into his treatment, the patient presented with bilateral lower extremity weakness. MRI of the spine revealed multiple enhancing nodules suspicious for leptomeningeal metastasis. He started a dexamethasone taper and underwent palliative radiation to the spine, with rapid improvement in strength within 1 week of completing radiation therapy and near complete resolution of symptoms within 3 weeks.

**Figure 1.**
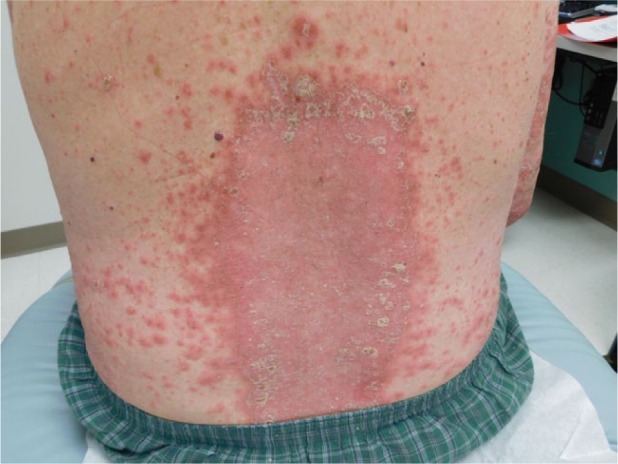
Sharply demarcated erythematous plaques with overlying scale on the back.

**Figure 2.**
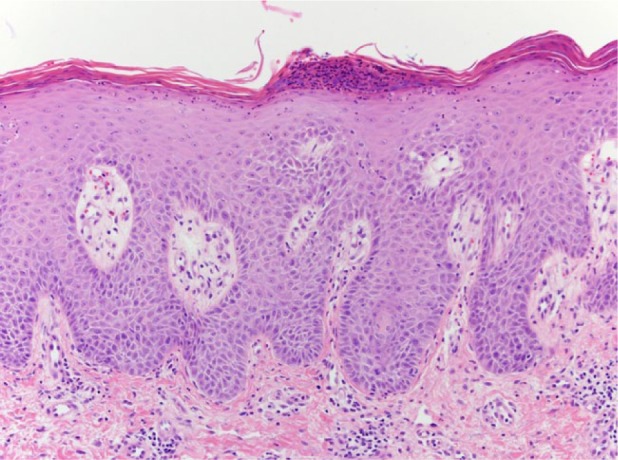
Regular psoriasiform epidermal hyperplasia with loss of the granular cell layer, parakeratosis, and Munro’s microabscesses (hematoxylin-eosin, 200×).

The patient then underwent 2 more doses of pembrolizumab followed by a restaging PET/CT, which revealed a complete response with interval resolution of the hypermetabolic lymphadenopathy and the osseous lesions. Physical examination, however, revealed diffuse scaly plaques on the trunk and the upper and lower extremities (Figure 1). This was determined to be a grade 3 toxicity, and pembrolizumab therapy was held. Punch biopsy of a lesion on the right leg demonstrated psoriasiform epidermal hyperplasia with parakeratosis and intraepidermal pustules confirming the diagnosis of psoriasis (Figure 2). He had no signs or symptoms of worsening psoriatic arthritis. The patient began therapy with acitretin and narrow band ultraviolet B phototherapy with substantial improvement in the psoriatic lesions.

As the patient had a significant tumor response to pembrolizumab therapy, it was restarted after 4 weeks, when the skin toxicity had improved to grade 1. The patient has since tolerated treatment well, without a psoriasis exacerbation.

## Case 2

D.N. is a 75-year-old Caucasian man with a history of malignant melanoma of the left shoulder resected in 2011, who presented with progressive dysphagia. An upper endoscopy revealed a gastric mass, and biopsy specimens demonstrated a poorly differentiated malignant neoplasm. Staging CT of the chest, abdomen, and pelvis demonstrated numerous pulmonary nodules bilaterally and scattered liver lesions consistent with metastatic disease. Biopsy of a liver lesion confirmed metastatic melanoma, negative for BRAF mutations. A brain MRI was negative for intracranial involvement. Pertinent medical history included acetylcholine receptor (AChR) autoantibody positive myasthenia gravis (MG) diagnosed at age 64. At that time, he had primarily ocular symptoms and AChR binding antibodies were positive at a titer of 2.1 nmol/L (normal range = 0.0-0.4 nmol/L). Over the years, his MG progressed and he developed other symptoms such as dysphagia, chewing fatigue, dyspnea on exertion, and neck and limb weakness. He responded well to prednisone and was eventually transitioned to mycophenolate mofetil. Just before the metastatic melanoma diagnosis, his MG was asymptomatic with mycophenolate mofetil 750 mg daily and pyridostigmine 60 mg 3 times daily.

For lack of a more robust treatment option, and after discussing the potential side effects with the patient and the neurologist, pembrolizumab was started at 2 mg/kg every 3 weeks. The dose of mycophenolate mofetil was decreased to 500 mg daily and pyridostigmine was continued unchanged. After his second dose, the patient developed symptoms of diplopia and worsening bilateral ptosis concerning for an exacerbation of MG. Pembrolizumab was held, and pyridostigmine was titrated up to 180 mg 3 times daily, along with prednisone 30 mg daily. The patient’s condition continued to deteriorate, and he was admitted to the intensive care unit with respiratory distress and worsening dysphagia, where he required noninvasive ventilation and gastrostomy tube placement. The patient’s AChR binding antibody titer was measured during his hospitalization and was elevated at 0.77 nmol/L (normal range <0.02 nmol/L).

His MG symptoms improved with 7 treatments of plasma exchange, 2 doses of intravenous immunoglobulin, and 4 doses of rituximab. On discharge from the intensive care unit, the patient underwent a restaging PET/CT scan, which showed resolving lung nodules, consistent with a partial response. Due to the grade 4 toxicity, pembrolizumab therapy was not resumed. Temozolomide was started at 75 mg/m^2^ daily. The patient was readmitted 1 month later for pneumonia secondary to aspiration and *Escherichia coli* bacteremia. He quickly succumbed to the infection.

## Discussion

Immune-related adverse events are well-recognized risks to checkpoint inhibitors such as pembrolizumab. However, patients with preexisting autoimmune disease were largely excluded from clinical trials evaluating checkpoint inhibitors due to concern that treatment would lead to unacceptable toxicities from exacerbations of underlying disease. Therefore, less is known about the significance of these exacerbations.

Both the patients we describe in this report experienced a tumor response to pembrolizumab therapy while simultaneously experiencing a flare of their autoimmune disease. For the patient in Case 1, we cannot entirely exclude the discontinuance of methotrexate as a contributing factor to his psoriasis flare; however, in our experience, the timing (3 months instead of the typical 6-8 weeks after discontinuing methotrexate) and severity of his flare (guttate in appearance and much different than his prior plaque type psoriasis) suggest that methotrexate withdrawal is unlikely to be the sole cause.

Checkpoint inhibitors induce irAE by nonspecific immunologic activation, decreasing the ability of effector T lymphocytes to discriminate between self and nonself.^6,7^ Some studies suggest that the development of irAE correlates with tumor response.^8,9^ Accordingly, we speculate that exacerbations of preexisting autoimmune conditions may also correlate with tumor response.

Isolated cases of MG were reported in studies evaluating PD-1 and PD-L1 inhibitors.^10-12^ Additionally, case reports of new diagnoses of MG secondary to checkpoint inhibitors have been described in the literature.^13-16^ There are also cases in the literature describing patients with preexisting MG who were treated with checkpoint inhibitors. One of these describes a patient with preexisting MG who developed an exacerbation while on the PD-1 inhibitor nivolumab for metastatic melanoma. After temporary cessation of nivolumab therapy, the exacerbation resolved, and a CT scan revealed a response in the metastatic lymph nodes.^17^ Another discussed a patient with advanced melanoma and preexisting MG who developed a severe exacerbation after 3 doses of pembrolizumab. He was reported to have stable disease at 4-month follow-up.^18^ Two published case reports describe patients with advanced melanoma experiencing exacerbations of preexisting psoriasis while on nivolumab.^19,20^ In one of those reports, the patient was described as experiencing a clinical response to nivolumab therapy.^19^

While there was initially concern among oncologists regarding the safety of using checkpoint inhibitors in patients with preexisting autoimmune disease, a recent retrospective review examining the use of the CTLA-4 inhibitor ipilimumab in such patients revealed that while ipilimumab therapy was associated with exacerbations of autoimmune disease, they were largely manageable with conventional immunosuppressive therapies.^21^ Additionally, one case report describes the use of immunotherapies in the treatment of metastatic melanoma in 2 patients with preexisting autoimmune diseases, and no exacerbations were observed.^22^ Of course, one must consider the potential morbidity from an exacerbation of underlying autoimmune disease prior to initiating checkpoint inhibitor therapy.

Anecdotally, clinicians have noted a link between autoimmunity and tumor response, such as the development of vitiligo after treatment with IL-2. In fact, Phan et al demonstrated an association between long-term immunologic side effects, especially vitiligo, and an antitumor response to IL-2 in patients with melanoma.^23^ These findings were validated in a meta-analysis that showed that patients who developed vitiligo while receiving immunotherapy had 2 to 4 times less risk of disease progression and death compared to patients without vitiligo.^24^ A study by Hua et al suggested a similar link between vitiligo and response to pembrolizumab therapy with 71% of patients with vitiligo experiencing a response to therapy versus 28% without vitiligo.^25^ In a recent study published by Sanlorenzo et al, 42% of patients treated with pembrolizumab developed cutaneous adverse events. These patients had a significantly longer progression-free survival than patients who did not develop cutaneous adverse events.^26^

Another study showed that administration of a CTLA-4 inhibitor in combination with an antimelanoma vaccine resulted in 14 patients developing grade 3 or 4 autoimmune toxicity with 36% of those patients experiencing a clinical response compared to 5% in those patients experiencing no autoimmunity.^8^ A single-institution study showed a significantly higher clinical benefit rate in patients on a CTLA-4 inhibitor who developed grade 3 or 4 irAE (60%) compared with those with grade ≤2 irAE (22%).^9^

The idea of an association between autoimmune toxicity and therapeutic response has long been debated. There are studies and case reports in the literature describing patients treated with IL-2 or interferon who were subsequently diagnosed with new cases of autoimmune conditions including diabetes mellitus and thyroid disease as well as a report of 2 patients with exacerbations of their underlying Crohn disease.^27-30^ While there was an association noted in some of these patients, development of autoimmune disorders was not consistently correlated with therapeutic response. Another case report describes a patient with renal cell carcinoma treated with IL-2 who subsequently developed MG, myositis, and insulin-dependent diabetes mellitus. Interestingly, the authors were able to determine retrospectively that the patient had preexisting autoantibodies against striated muscle and insulin but without any clinical manifestations. He remained on chronic immunosuppression after discontinuation of IL-2. The authors note that while he did have regression of his metastatic cancer, the continuing need for immunosuppression may have negated this effect.^31^

The mechanism by which checkpoint inhibitors trigger autoimmunity is incompletely understood, and may be related to dysregulation of a preexisting immune response to self-antigens, which had been formerly contained by immune checkpoints.^32^ Additionally, proliferation of effector T cells combined with impairment in regulatory T cells and dysfunctional interactions with antigen presenting cells may play a role.^33^ Interestingly, while the 2 autoimmune disorders we presented have very different clinical manifestations, both involve CD4+ T cells. Psoriasis in particular is thought to be primarily a T-cell-mediated process, in which there is toxic injury to the epidermis resulting in the characteristic skin plaques and hyperproliferation.^34^ Accordingly, advances in therapy have been made through agents that block T cell functions.^35^ MG is driven by CD4+ T cells that are AChR specific, activating B cells, which, in turn, produce AChR autoantibodies.^36^ While the precise pathologic basis of autoimmunity is unknown, research is ongoing to identify biomarkers that predict response to checkpoint inhibitor therapy as well as predict for development of irAE.^37^

## Conclusion

While intriguing, further investigation is needed into the relationship between irAE and response to immunotherapy. As the use of checkpoint inhibitors continues to broaden and biomarkers of response are discovered, perhaps the speculative link between autoimmunity and response to therapy will be elucidated, leading to more informative discussions with patients.
